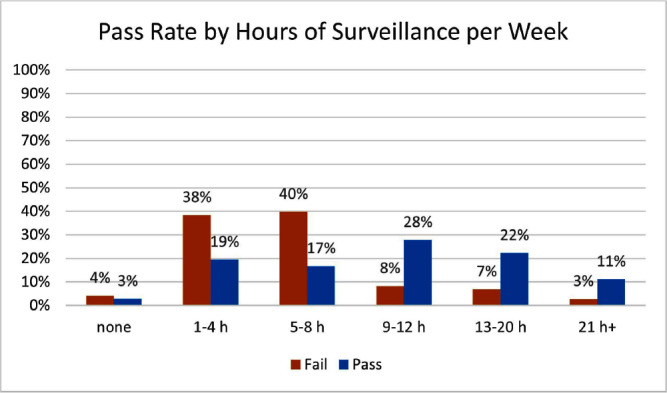# Ensuring Accuracy: Making the Case for Inter-rater Reliability in Hospital Acquired Infection Surveillance

**DOI:** 10.1017/ash.2024.324

**Published:** 2024-09-16

**Authors:** Kelly Holmes, Mishga Moinuddin, Sandi Steinfeld

**Affiliations:** Infection Prevention & Management Associates

## Abstract

**Background:** Infection preventionists (Ips) have self-reported surveillance as the most time-consuming job task (1,2). APIC’s MegaSurvey 2020 reported that 60% of Ips consider themselves proficient or expert within this competency domain (2). Accurate coding of health care acquired infections is critical to identifying epidemiologically significant events, using data to improve practice, and compliance with state and federal mandated CMS reporting (3,4). Validated case study scenarios were distributed to infection preventionists to better understand how experience level and time spent performing surveillance affects interrater reliability (IRR) in applying the National Healthcare Safety Network (NHSN) surveillance definitions (5,6). **Methods:** Case study scenarios determined to have high item discrimination were added to an online test bank and distributed annually to Ips of varying experience levels and care settings as part of a mandatory training program (5). The test bank currently consists of forty-two validated questions. Each year, the participants receive approximately thirty questions, including twenty randomly selected from the test bank and ten beta scenarios under development. Only validated test bank scenarios are used to calculate the passing score of 85%. Participants are blinded to which questions are test bank scenarios versus beta scenarios. Additional information was gathered at the beginning of the test to determine CIC status, years of experience, and weekly hours spent doing surveillance. Data was analyzed for passing score on the first attempt for testing years 2019, 2021, 2022, and 2023. **Results:** Thirty-six Ips passed the IRR test on the first attempt (33%). Of those who passed on the first attempt, twenty-nine (81%) were certified and twenty-two (61%) reported at least nine hours a week performing surveillance. Of the seventy-three Ips (67%) who did not pass on the first attempt, thirty were certified (41%) and sixty (82%) reported performing surveillance for 8 hours or less per week. **Conclusion:** The first-time pass rate for certified and non-certified Ips was 33%, markedly lower than the self-reported proficiency rate of 60%. The majority of Ips who passed on the first attempt were certified and spent at least nine hours per week performing surveillance. certified and non-certified Ips who did not regularly perform surveillance as part of their weekly job tasks were less likely to pass the test on the first attempt. Given the first-time pass rate among all participants is below optimal, establishing inter-rater reliability systems and ongoing surveillance education for Ips is crucial to ensure accuracy of publicly reported data.

**Figure:**
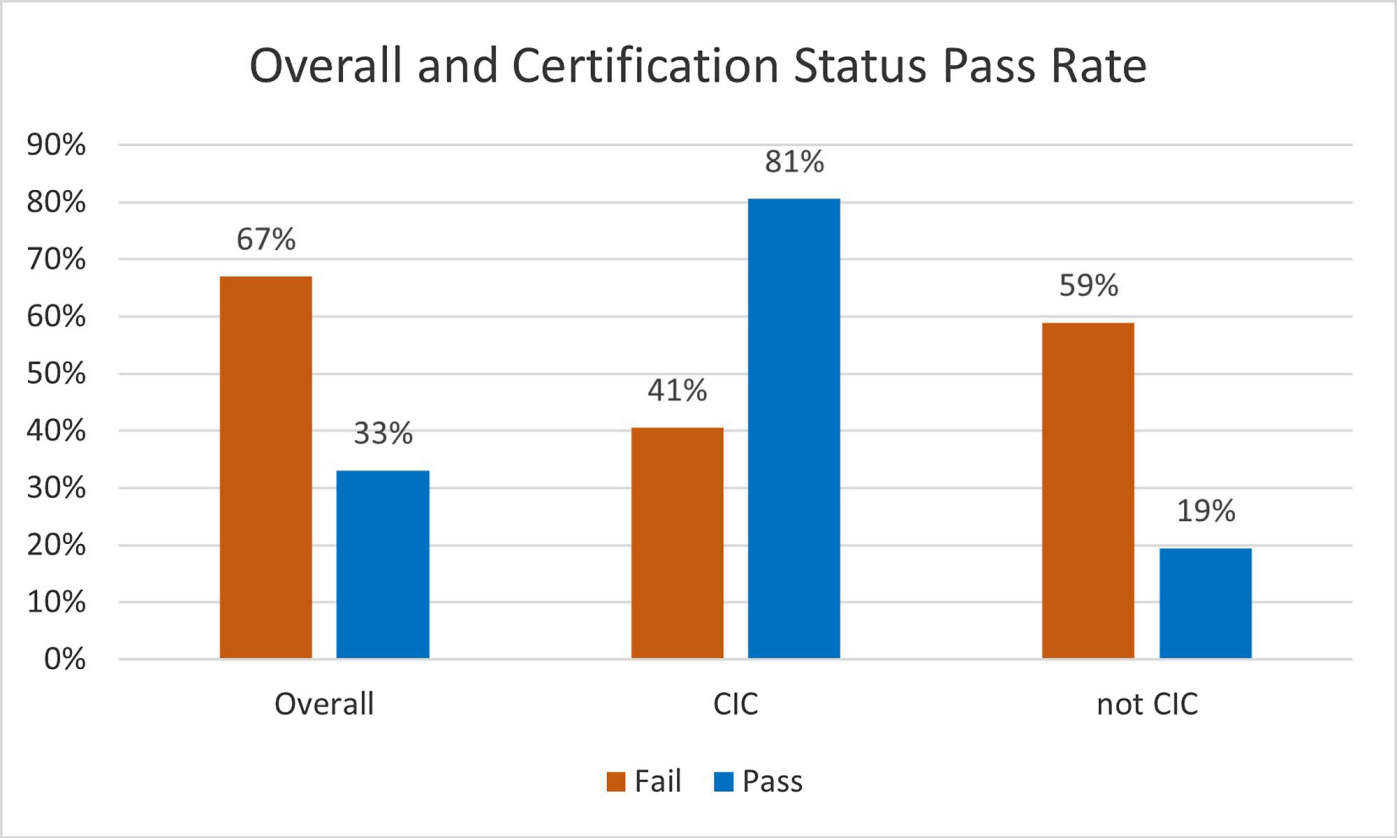

**Figure:**